# Chromatin Dynamics during Lytic Infection with Herpes Simplex Virus 1

**DOI:** 10.3390/v5071758

**Published:** 2013-07-16

**Authors:** Kristen L. Conn, Luis M. Schang

**Affiliations:** 1 Department of Biochemistry, Li Ka Shing Institute of Virology, University of Alberta, Edmonton, AB T6G 2H7, Canada; 2 Department of Medical Microbiology and Immunology, University of Alberta, Edmonton, AB T6G 2E1, Canada

**Keywords:** Herpes simplex virus 1, chromatin, silencing, nucleosome, histone, fluorescence recovery after photobleaching (FRAP)

## Abstract

Latent HSV-1 genomes are chromatinized with silencing marks. Since 2004, however, there has been an apparent inconsistency in the studies of the chromatinization of the HSV-1 genomes in lytically infected cells. Nuclease protection and chromatin immunoprecipitation assays suggested that the genomes were not regularly chromatinized, having only low histone occupancy. However, the chromatin modifications associated with transcribed and non-transcribed HSV-1 genes were those associated with active or repressed transcription, respectively. Moreover, the three critical HSV-1 transcriptional activators all had the capability to induce chromatin remodelling, and interacted with critical chromatin modifying enzymes. Depletion or overexpression of some, but not all, chromatin modifying proteins affected HSV-1 transcription, but often in unexpected manners. Since 2010, it has become clear that both cellular and HSV-1 chromatins are highly dynamic in infected cells. These dynamics reconcile the weak interactions between HSV-1 genomes and chromatin proteins, detected by nuclease protection and chromatin immunoprecipitation, with the proposed regulation of HSV-1 gene expression by chromatin, supported by the marks in the chromatin in the viral genomes and the abilities of the HSV-1 transcription activators to modulate chromatin. It also explains the sometimes unexpected results of interventions to modulate chromatin remodelling activities in infected cells.

## 1. Introduction

Herpes simplex virus 1 (HSV-1) establishes lytic infections in most cells, but latent infections in neurons *in vivo*. During lytic infections, the structural virion protein VP16 indirectly binds to the ‘TAATGARAT’ sequences in the promoters of the “immediate-early” (IE) genes in a complex with cellular HCF-1 and Oct1 to activate IE gene transcription [1,2,3,4,5,6]. Two IE proteins, ICP4 and ICP0, then activate transcription of ‘early’ (E) and ‘late’ (L) genes. The mechanisms of transcription activation by ICP0 and ICP4 are not yet fully characterized, but require no binding to specific DNA sequences [7,8]. It is widely accepted that at least ICP0 activates HSV-1 transcription, mostly by counteracting not yet fully characterized cellular defenses [9,10,11,12]. ICP4 activates transcription through interaction with components of the RNA polymerase II transcription machinery, such as the TFIID and mediator complexes. E proteins replicate HSV-1 DNA, whereas the L genes encode structural and other proteins required for the assembly of infectious virions. In contrast to lytic infections, transcription (except for the LAT locus), HSV-1 DNA replication, and production of new virions, are all inhibited during latency. HSV-1 mutants in the transcription activators ICP4, ICP0, and VP16 are also transcriptionally silent (*i.e.*, ‘quiescent’) in fibroblasts [13,14,15,16,17,18]. Quiescent infections can also be established in certain neuronal cultures under specific conditions [19,20,21,22,23].

Transcription, replication, and encapsidation of the approximately 150 kbp double stranded DNA HSV-1 genome all occur in the nucleus. Physiologically, nuclear DNA is organized in chromatin, a chain of nucleosomes. Each nucleosome is composed of 165 bp of DNA wrapped 1.46 times around an octamer of core histones H2A, H2B, H3 and H4. The core nucleosome is bound by linker histone H1, for a total of 200 bp and ~2 turns. The linear chain of nucleosomes is then folded into complex higher order structures to form the chromatin fibers. Chromatin is essential to compact the long eukaryotic genomic DNA to fit within the cell nucleus. For example, the more than 1m of human genomic DNA is packaged within the human nuclei (which are typically ~7 µM in diameter). It also regulates DNA accessibility and, consequently, all processes requiring access to nuclear DNA. This regulation is mediated largely by histone post-translational modifications (PTM) and the different variants. The amino-terminal tails of the core histones are extensively post-translationally modified. These PTM provide docking sites for proteins that regulate transcription (the proposed “histone code” [24,25,26,27,28]) and regulate nucleosome stability [29,30]. The different histone variants also regulate DNA accessibility. One variant of histone H3, CENP-A, for example, is particularly enriched in non-transcribed centrosomes [31]. Another, H3.3, is enriched in transcribed chromatin, perhaps because it forms unstable nucleosomes with a variant of histone H2A, H2A.Z [32,33,34]. Linker histones also regulate accessibility to the nucleosomal DNA [35,36,37,38,39,40]. Transcribed genes have lower H1 density than silenced ones [41,42,43]. Most nucleosome turnover occurs during transcription, DNA repair, and DNA replication, when the nucleosomes are disassembled to allow access to the DNA. Subsequently, nucleosomes are reassembled to maintain chromatin integrity. Nucleosome dynamics are thus important in the regulation of the processes requiring access to cellular DNA.

We will focus on HSV-1 to discuss the recent evidence strongly implicating nucleosome dynamics in the regulation of viral gene expression, and perhaps, DNA replication. The overarching model is that the infected cells attempt to silence the infecting genomes by mobilizing histones away from their chromatin to assemble silencing chromatin on the viral genomes. However, viruses have evolved proteins that counteract this silencing, to allow viral replication. These proteins therefore act as (indirect) activators of viral transcription.

## 2. Results and Discussion

### 2.1. The Chromatinization of HSV-1 Genomes in Lytically Infected Cells, an Apparent Paradox

The first evaluations of the nucleoprotein complexes containing HSV-1 DNA were performed in the context of the then current understanding that chromatin served mostly structural functions. Gibson and Roizman showed in 1971 that encapsidated HSV-1 genomes are complexed with enough spermine to neutralize 40% of the negative charges [44]. Mouttet and colleagues first described that nuclear HSV-1 genomes are far more accessible to micrococcal nuclease (MCN) than the DNA in the cellular chromatin [45], as confirmed later [18,46,47,48,49,50]. Muggerridge, in Fraser’s group, tested the chromatinization of HSV-1 genomes in infected mice [51]. During acute infection, the viral genomes were far more accessible to MCN than the cellular chromatin [51]. However, Deshmane, also in Fraser’s group, showed that the HSV-1 DNA in latently infected neurons was only as accessible as the cellular DNA [52]. Similar apparent regular chromatinization was later observed for quiescent HSV-1 genomes [53], chromatinization which was disrupted during reactivation [54]. The overall classic conclusion from all these results was that HSV-1 genomes were not regularly chromatinized in lytically infected cells, but were regularly chromatinized during latency or quiescence.

When chromatin was found to regulate gene expression, new techniques were developed to study chromatin in this context. The potential roles of chromatin in the regulation of HSV-1 gene expression were re-visited with these techniques. The pioneering work from Dr. Bloom’s group showed that latent HSV-1 genomes are associated mostly with chromatin bearing PTM of silenced genes [55], except for the transcriptionally active promoter of the LAT gene [56]. In contrast, the promoter of the ICP0 gene is associated during reactivation with chromatin bearing PTM of transcribed genes [57]. Independent results from Dr. Knipe’s group are mostly consistent with those from Dr. Bloom’s group, although virus and mouse strain-specific differences became apparent [58,59]. All of these results were fully consistent with the classic models that latent HSV-1 genomes were regularly chromatinized.

The seminal papers published in 2004 from Triezenberg’s, Fraser’s and Berger’s groups demonstrated that HSV-1 genomes surprisingly also co-immunoprecipitate with histones in lytically infected cells [50,60]. The histones associated with HSV-1 DNA appear to regulate viral gene expression. Highly transcribed HSV-1 genes associate with histones bearing PTM associated with transcription, whereas non-transcribed genes associate with histones bearing PTM associated with silenced genes [60,61,62] (although there are some exceptions [63]). The specific histone variants were also consistent with the transcription status in that histone H3 variant H3.3 associated preferentially with transcribed genes [64]. In cells with HCF-1 knocked down, HSV-1 DNA was not transcribed and predominantly in chromatin with marks associated with silencing [65]. Consistently, recruitment of histone acetylases [60], methyltransferases (Set1) [66] or demethylases (LSD1) [67] to IE promoters by VP16 appears required to activate transcription [68] (but see also [69]).

**Figure 1:**
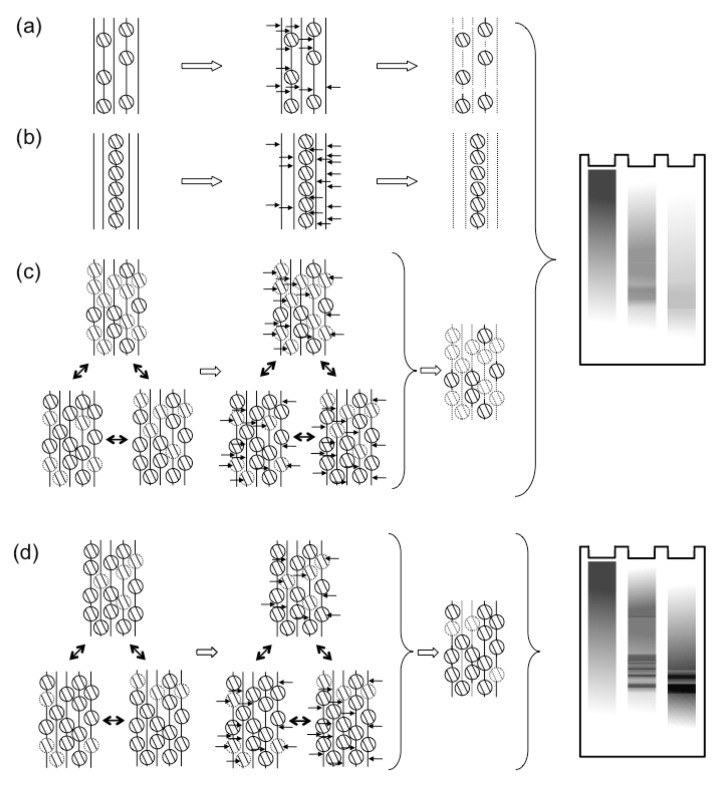
The dynamics of HSV-1 nucleosomes in lytic or latent infections. (**a**, **b**) Classic models proposed that most HSV-1 genomes were not properly chromatinized in lytic infections. Either all genomes were proposed to have only sporadic nucleosomes, or some genomes were proposed to be chromatinized, whereas most were proposed to not associate with nucleosomes. Both models would result in the observed protection from MCN digestion to mostly fragments of variable sizes, which show as a “smear” in Southern blots. In contrast, latent genomes were proposed to be normally chromatinized. In these classic models, the difference between lytic and latent viral genomes was qualitative (*i.e.*, non‑chromatinized or chromatinized, respectively). (**c**) More recent evidence points to the HSV-1 genomes forming unstable nucleosomes, which unbind during the digestion, failing to protect the viral DNA. (**d**) In contrast, latent or quiescent HSV-1 genomes are in far more stable nucleosomes, which protect their DNA from MCN digestion, resulting in the typical “nucleosome ladder”. According to the more recent evidence, the difference between lytic and latent HSV-1 genomes is quantitative (*i.e.*, different degree of nucleosome stability).

These and other results suggesting that HSV-1 transcription was regulated by chromatin were difficult to reconcile with the earlier results of MCN digestions. Regularly chromatinized cellular DNA is digested by MCN to fragments of sizes of multiples of nucleosome DNA. In contrast, the HSV-1 DNA in lytically infected cells is digested to fragments of heterogeneous sizes (for examples, see [18,45,46,47,48,49,50]). Nuclear HSV-1 DNA is also digested much faster than cellular DNA (although also much slower than naked DNA). Only much smaller percentages of HSV-1 than cellular DNA consistently co-immunoprecipitate with histones (for example, see [50,60,61,62,65]; for a notable exception, at a low multiplicity of infection, see [70]). A most common interpretation was that the HSV-1 DNA in lytically infected cells had low nucleosome occupancy, with long “linker” non‑nucleosomal DNA of variable sizes between nucleosomes [54,63,70,71] (Figure 1a). This model could account for the variable sizes of the protected fragments and the small percentage of HSV-1 DNA protected to nucleosome sizes or co-immunoprecipitated with histones. However, it is difficult to reconcile with the models in which chromatin regulates HSV-1 transcription [9,72,73,74]. Low-density nucleosomes would not be expected to act as a barrier to impede transcription, neither to cooperatively recruit transcription activators. Alternatively, only a small subpopulation of the viral genomes could be regularly chromatinized, whereas the majority would not be associated with any histones (Figure 1b). The former would be protected to nucleosome sizes and co-immunoprecipitated with histones; the latter, randomly cleaved and not co-immunoprecipitated. This model is compatible with the proposed chromatin regulation of gene expression only if the specific subset of properly chromatinized HSV-1 genomes contained all the biologically relevant ones. Interestingly, only a subpopulation of all the genomes of a related alpha-herpes virus that infect a cell start successful replication cycles [75]. However, there is no evidence that the biologically active genomes are fully chromatinized and the inactive ones are not.

Mixed models, in which some genomes would have higher nucleosome density than others, have the same limitations of the two models discussed. The simplest prediction of any combination of these models is that the association of differentially transcribed HSV-1 genomes with histones bearing PTM of transcribed or non-transcribed chromatin is functionally irrelevant. In apparent support of this prediction, depletion of certain histone lysine acetyltransferases (KAT) and other chromatin modifiers, or infections of cells lacking specific chromatin remodellers, had no obvious effects on HSV-1 transcription or replication [69]. However, the depletion of others or of the histone chaperones that assemble chromatin, affected HSV-1 transcription or replication [61,64,76].

Another line of evidence supports the models proposing that chromatin regulates HSV-1 transcription. While it was still generally accepted that chromatin played no role in HSV-1 transcription, all three major HSV-1 transcription activators, VP16, ICP0, and ICP4, were surprisingly found to be capable of (indirectly) inducing chromatin modifications. As discussed in detail later, ICP0 interacts with several histone deacetylases [77,78,79], destabilizes CENP-A [80] and decreases the levels of ubiquitinated H2A variants [81]. ICP0 and ICP4 together disrupt silencing of cellular facultative heterochromatin, although the biological importance of this activity remains unclear [82,83]. ICP4 functionally interacts with two chromatin architectural proteins, HMGA and B [84,85], although the biological importance of these interactions, if any, also remains unknown. VP16 also has chromatin remodelling activities. Being a classic model for activators of eukaryotic transcription, VP16 was used to study transcription regulation by chromatin [86]. In this artificial model, a large number of binding sites for the lac repressor were recombined in the cellular genome. The acidic carboxy-terminal transcription activation domain of VP16 was then expressed as a fusion protein with the DNA binding domain of the lac repressor (and an EGFP tag) in the absence of any other HSV-1 factor. Under these conditions, the VP16 activation domain promoted large-scale chromatin decondensation (spanning several megabases) [86]. Decondensation preceded the activation of transcription [87]. The relevance of these VP16 activities for HSV-1 remained unclear at the time, when chromatin was assumed to play no role in the regulation of HSV-1 gene expression.

### 2.2. Chromatin and Histone Dynamics Are Altered in Cells Lytically Infected with HSV-1

#### 2.2.1. HSV-1 DNA Is in Unstable Nucleosomes during Lytic Infections

The abilities of the HSV-1 transcription activators to modulate chromatin are consistent with the models proposing that chromatin generally inhibits HSV-1 gene expression in lytic infections [9,72,73,74,88]. Also in support of such models, deletion of VP16, ICP0, and ICP4 results in quiescent infections [13,14,15], in which the HSV-1 DNA is in chromatin with silencing PTM [13,53]. Quiescent infections of cultured neurons are also characterized by silencing PTM on the viral nucleosomes, which are disrupted when VP16 is recruited to the viral genomes during reactivation [23]. However, the histone occupancy of HSV-1 genomes, as tested by ChIP or MCN accessibility assays, appears far too low for the standard mechanisms of transcription regulation by chromatin [69,89]. ChIP assays, however, do not detect unstable cellular nucleosomes [34], which are enriched in highly transcribed genes. Likewise, MCN assays are based on protection and, consequently, are not well suited to identify highly dynamic DNA-protein interactions.

As of 2010, there was an apparent inconsistency in the data regarding the chromatinization of HSV‑1 genomes during lytic infections. To explore it, we analyzed the chromatin from HSV-1 infected cells by classical biophysical chromatin fractionation [47]. HSV-1 DNA forms large branched structures during replication. We therefore first restricted the nuclear DNA with BamH1, to release fragments of chromatin or naked DNA of sizes that can be resolved in sucrose gradients. Deproteinized HSV-1 DNA added to nuclei of HSV-1 infected cells before restriction fractionated to the very top fractions almost exclusively, as expected for naked DNA. Cellular DNA fractionated to lower fractions, as expected for chromatinized DNA. Most surprisingly, the nuclear HSV-1 DNA fractionated almost exclusively like regularly chromatinized cellular DNA, not like deproteinized HSV-1 DNA. Consistently with the classic results, however, only a minority of the HSV-1 DNA fragments released by standard MCN digestions fractionated as chromatinized DNA. Nonetheless, basically all HSV-1 DNA released in the “soluble chromatin” fraction (*i.e.*, the chromatin not pelleted in 20 minutes at 8,000 g) fractionated as cellular mono- to di-nucleosomes after multistep fractionations through differential centrifugation, sucrose gradient, and size exclusion chromatography. Most intriguingly, the percentage of HSV-1 DNA that was released in complexes migrating as mono- to di-nucleosomes did not increase with MCN digestion time. As expected, longer digestions resulted in higher percentages of cellular DNA released in complexes migrating as mono- to di-nucleosomes. Among the possibilities, the released HSV-1 DNA might have been protected in unstable complexes. These unstable complexes would allow their DNA to be further digested, essentially to nucleotides, during longer digestions. Meanwhile, new complexes of mono- to di-nucleosome size would be released from the “insoluble chromatin” fraction (*i.e.*, the chromatin pelleted in 20 minutes at 8,000 g), replenishing the pool of mono- to di-nucleosome sized complexes in the “soluble chromatin” fraction.

To test this model, we designed a variation of the standard MCN protection assays [47]. The MCN digestion was performed while the soluble and insoluble chromatins were being fractionated by centrifugation. The digestion was stopped every 5 minutes, the released soluble chromatin was collected and the MCN in it was immediately quenched to prevent further digestion of the released complexes. New MCN was added to the insoluble chromatin and the process was repeated. After nine [47] or six [46] repeats, all the soluble chromatin collected was pooled and fractionated by sucrose gradient. Under these limiting digestions, most HSV-1 DNA was released into the soluble chromatin in fragments of sizes of multiples of the nucleosome DNA (Figure 1c). In fact, the fractionation of the HSV-1 DNA complexes was indistinguishable from that of the cellular DNA complexes [47]. The fractionated mono- to di-nucleosome sized nucleoprotein complexes were next cross-linked before re-digesting them with MCN [47]. The complexes containing cellular DNA were equally protected with or without cross-linking, as expected for stable complexes. In contrast, the complexes containing HSV-1 DNA were far better protected from the MCN re-digestion after cross‑linking [47], consistent with their proposed instability (Figure 1c).

Nucleosomes are disassembled during DNA replication, to allow the progression of the replication fork, and are reassembled behind it. The HSV-1 nucleoprotein complexes could very well be unstable only because of the fast rate of HSV-1 DNA replication. However, the HSV-1 nucleoprotein complexes were still far more unstable than cellular nucleosomes when HSV-1 DNA replication was inhibited with phosphonoacetic acid (PAA) [46]. The degree of instability was affected by PAA, however, indicating that HSV-1 DNA replication does modulate the dynamics of the HSV-1 nucleosomes to some extent.

Two different patterns of HSV-1 nucleosome stability were observed at the earliest time that could be studied (1h after removing inocula). At these early times, the HSV-1 DNA was in very inaccessible nucleoprotein complexes in half of the experiments, or in the unstable complexes in the other half [46]. The highly resistant HSV-1 DNA observed at these early times (in half of the experiments) may well be HSV-1 DNA that has been mostly silenced by the cell and not yet desilenced by the virus. Alternatively, it could be DNA that is not yet decapsidated, or a mix of encapsidated and silenced DNA. However, HSV-1 DNA in naked capsids is fully resistant to MCN [90], whereas most of the so-called inaccessible nuclear HSV-1 DNA is eventually digested [47].

#### 2.2.2. Histone Dynamics during Lytic Infections

As discussed, the interactions between histones and HSV-1 DNA are far more dynamic than those with cellular DNA. The DNA in the infecting virions amounts to only a fraction of the cellular DNA under common culture conditions. Assuming 10 infectious virions per cell and a reasonable 1:100 ratio of infectious to non-infectious virions, the total amount of HSV-1 DNA infecting a nucleus would amount to 2.5% of the cellular DNA if all genomes entered into the nucleus. Not all do, and the number of virions infecting each cell *in vivo* is estimated to be lower. However, HSV-1 DNA replication is exponential, and the total amount of viral DNA would still reach up to a significant proportion of the total nuclear DNA at later times after infection if 10 viral genomes replicated 1,000-fold each.

**Figure 2:**
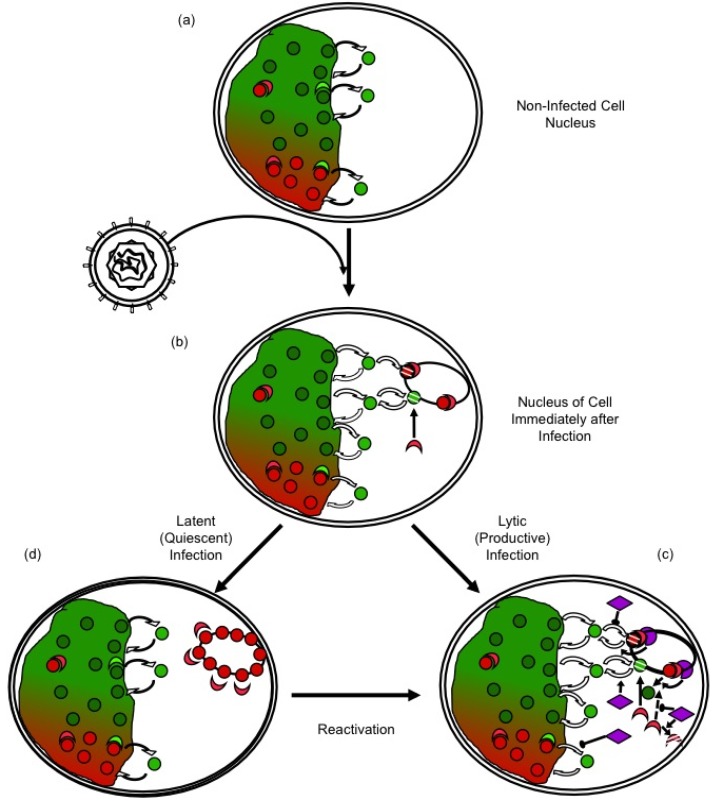
Regulation of HSV-1 gene expression by chromatin silencing and anti-silencing. (**a**) In the nuclei of non infected cells, histones (red and green circles) unbind from chromatin, diffuse through the nucleus and rebind to a different site. The diffusing histones are bound to histone chaperones in the “free pool”. Histones exchange in both transcribed (green) and silenced (red) chromatin, but at different rates. Chromatin modifying enzymes (crescent shapes) modulate histone dynamics by modulating affinity for chromatin, DNA, or other proteins. (**b**) Immediately after nuclear entry, HSV-1 genomes are complexed by the cell in silencing chromatin (red), to which further silencing chromatin modifying proteins are recruited (red crescent shapes). (**c**) In productive infections, HSV-1 proteins (purple) mobilize histones away from the viral genomes, by indirectly modulating their PTM, availability, localization, or interaction with other proteins, and recruiting activating chromatin modifying proteins (green crescents). (**d**) Viral genes are then transcribed and the genomes are replicated. In latent or quiescent infections, the initial silencing chromatin is not disrupted efficiently, leading to the formation of (almost) fully silenced chromatin, which must be disrupted for reactivation to occur.

Most cells are not infected in S-phase when most core histone synthesis occurs, and infection inhibits histone synthesis [91,92,93]. There is a population of histones at any given time in the process of exchanging between different DNA binding sites (Figure 2a). These exchanging histones are momentarily not in nucleosomes and could therefore be used to chromatinize the infecting viral genomes. However, such sequestration of free histones would alter the equilibrium, indirectly promoting further release of histones from the cellular chromatin (Figure 2b). Considering the magnitude of the pool of the HSV-1 DNA at later times, the normal pool of the free histones would be insufficient to chromatinize all HSV-1 DNA. There is of course a much larger pool of histones in the cellular chromatin, but these histones would have to first be mobilized away from the cellular chromatin to be available (Figure 1c). Moreover, any histones in the particularly unstable HSV-1 chromatin would undergo chromatin exchange at a much faster rate than those in the cellular chromatin.

We therefore evaluated whether histone dynamics were altered in infected cells. Such dynamics can only be analyzed in intact nuclei. We therefore used fluorescence recovery after photobleaching (FRAP) [94], which had already been used to describe histone dynamics in non infected cells [95,96,97]. The highly mobile linker histone H1 was further mobilized in infected cells in a variant- and multiplicity-dependent manner [94]. The mobilization of the variant H1.2, which is widely expressed in all cells in which HSV-1 replicates, was evaluated in detail. Mobilization required the presence of transcriptionally capable HSV-1 genomes in the nucleus. It did not require VP16 or ICP0, but it was enhanced by VP16. It was surprisingly not affected by HSV-1 DNA replication, in that PAA did not affect it. At a given multiplicity of infection, H1.2 was mobilized to a larger degree in U2OS cells, which functionally complement for defects in VP16 and ICP0 [94], than in Vero cells, which do not. U2OS appear to be defective in their ability to mobilize all histones in response to infection. This inability may facilitate the replication of mutants defective in proteins that counteract silencing.

H1 mobilization may or may not reflect the faster core histone exchange predicted by the instability of the HSV-1 nucleosomes. It would not provide the core histones required to assemble HSV-1 nucleosomes, either. We therefore evaluated next the dynamics of core histones. We started with histones H2B and H4 [98], which have no variants that could be differentially mobilized. They also are components of each of the two tetramers within the nucleosome. Both H2B and H4 were mobilized in infected cells. Their free pools had already increased at four hours after infection, and continued to increase for another three hours (reaching approximately 50% over the free pools in mock infected cells). 

The mobilization of H2B was analyzed in further detail. Its rate of fast chromatin exchange was slower in infected cells [98]. H2B is therefore likely mobilized away from cellular chromatin by preferentially inhibiting the re-binding of unbound H2B, not by promoting its release. H2B mobilization required no VP16 or ICP0, but both VP16 and ICP0 enhanced it [98]. Mobilization in the absence of VP16 and ICP0 in complementing U2OS cells did not decrease the rate of fast chromatin exchange [98]. Mobilization in U2OS cells in the absence of ICP0 alone even decreased the rate of fast chromatin exchange [98]. These results suggest that VP16 is the primary modulator of H2B fast chromatin exchange, at least under conditions of viral replication. Moreover, the pool of free H2B increased to a larger extent in Vero cells infected with the ICP0 null mutant n212 [98], suggesting that ICP0 induces the degradation of the mobilized histones. This potential role of ICP0 would be consistent with its many other ones inhibiting cellular defenses against viral gene expression [9].

Most surprisingly, H2B mobilization required no HSV-1 DNA replication (or late proteins) [98]. It did, however, require nuclear and transcriptionally capable HSV-1 genomes (although not their actual transcription). Mobilization was not dependent on relative histone levels, and the histones were mobilized throughout the population of infected cells [98].

The H3 variant H3.1 associates with HSV-1 genomes only after the onset of HSV-1 DNA replication, whereas the H3.3 variant associates with them, both before and after. The evidence to date suggests that histones are mobilized away from the cellular chromatin to silence HSV-1 genomes, but that HSV-1 proteins counteract this silencing by further mobilizing histones. If this model was correct, then the mobilization of histones H3.1 and H3.3 would be differentially affected by inhibition of HSV-1 DNA replication. Our ongoing experiments suggest that this is indeed the case. Inhibition of HSV-1 DNA replication by PAA drastically increases the pools of free H3.1, but not those of free H3.3 [99]. These results suggest that H3.1 is mobilized away from cellular chromatin immediately after infection, but it is assembled into viral chromatin only during HSV-1 DNA replication. Another testable prediction of the model is that specific HSV-1 proteins should induce enhanced histone mobilization, and that these proteins should be involved in the regulation of HSV-1 gene expression (Figure 1c). We are also working to test this prediction.

### 2.3. Potential Mechanisms of Regulation of Nucleosome Dynamics in HSV-1 Infected Cells

The regulation of nucleosome dynamics in infected cells is not yet fully understood. The same cellular chromatin proteins, chromatin-modifying enzymes, and chromatin-remodelling complexes regulate viral and cellular chromatin dynamics. It is consequently difficult to distinguish between cellular and viral nucleosome dynamics, or to identify the processes that regulate the turnover of the nucleosomes on the viral genome among the background turnover of the nucleosomes on the cellular genome.

The HSV-1 genomes in virions have no associated histones [62] and enter the nucleus compacted with spermine [44]. They must then be decondensed and assembled into chromatin for the cellular transcription proteins to access them. These processes may be concurrent or sequential. Meanwhile, cellular antiviral responses target repressive proteins to viral genomes [100,101], promoting the assembly of silencing heterochromatin [73]. The histones in the HSV-1 chromatin immediately after infection consequently bear PTM characteristic of heterochromatin [68,72,102,103,104], as do those associated with latent or quiescent genomes [13,53,55]. In lytic infections, however, these early HSV-1 nucleosomes are promptly modified with PTM characteristic of euchromatin. These PTM may cause the instability of the HSV-1 nucleosomes [46,47] and the increase in the overall histone exchange rates [94,98]. Conversely, the rapid turnover of HSV-1 nucleosomes may in itself impair the repressive PTM, and the recruitment of repressive heterochromatin proteins. Increased nucleosome dynamics also directly facilitates access to HSV-1 DNA, fostering transcription. The instability and dynamic nature of the viral chromatin may in itself inherently counteract genome silencing.

The cellular antiviral responses would thus be expected to decrease the dynamics of HSV-1 chromatin, while increasing those of cellular chromatin to make chromatin proteins available to silence the viral genomes (Figure 2b). Conversely, HSV-1 proteins would be expected to increase the dynamics of the viral nucleosomes, while inhibiting those of cellular chromatin to minimize the availability of silencing proteins (Figure 2c). If the cellular antiviral responses generally mobilize histones away from the cellular chromatin, then an initial outcome of the infection would be a generalized increase in chromatin dynamics. Indeed, histones are mobilized upon nuclear entry of HSV-1 genomes [94,98]. Mobilized cellular repressive proteins are also likely targeted to the viral genomes, in that HSV-1 proteins inhibit this targeting [9]. HSV-1 proteins also recruit activating chromatin proteins and modifying complexes (e.g., KATs or chromatin remodellers) [60,105,106]. This recruitment directly stimulates viral nucleosome turnover and facilitates transcription. Moreover, it also isolates such complexes from the cellular chromatin, indirectly decreasing cellular nucleosome turnover and restricting the availability of repressive proteins. If cellular histones or chromatin-modifying proteins could not be mobilized away from cellular chromatin, then repressive proteins would be less available to silence the HSV-1 genomes. Then, it would be far less critical for HSV-1 to be able to counteract silencing.

#### 2.3.1. Mechanisms of Regulation of Nucleosome Dynamics

The stability and turnover of cellular nucleosomes are physiologically affected by several mechanisms, including: *(i)* the histone variants in the nucleosomes, *(ii)* their PTM, and *(iii)* the repositioning or eviction of nucleosomes by ATP-dependent chromatin remodelling complexes. These mechanisms most likely also modulate viral nucleosome turnover and stability.

***Histone variants.*** Of all core histone variants, only the H3 variants H3.1 and H3.3 have been analyzed in HSV-1 chromatin [64]. Both are mobilized early during infection [99]. However, H3.3 is preferentially assembled into the viral chromatin at this time, whereas H3.1 is only assembled into it after the initiation of viral DNA synthesis [64]. The similar mobilization and differential assembly of H3.3 and H3.1 into HSV-1 chromatin indicates the specificity of nucleosome turnover and assembly during infection.

The endogenous free pool of H3.3 is increased in infected cells by mobilization of H3.3 from cellular chromatin [99]. Physiologically, H3.3 is assembled into cellular chromatin by three histone chaperones: HIRA, hDaxx, and DEK [107,108,109]. HIRA and hDaxx also participate in the assembly of HSV-1 chromatin [100]. Knockdown of HIRA decreases H3.3 interaction with HSV-1 genomes [64]. If HSV-1 nucleosomes strictly opposed transcription, then reduced H3.3 occupancy should increase viral transcription. Surprisingly, knockdown of HIRA decreases HSV-1 transcription [64]. HIRA knockdown also affects cellular chromatin dynamics, however, and could thus hinder the mobilization of H3.3 away from cellular chromatin. It would consequently decrease the pool of H3.3 available for silencing viral genomes. This decrease may affect the decondensation of decapsidated HSV-1 genomes, or their proper assembly into transcription competent chromatin. HIRA may also be important for viral nucleosome turnover during transcription (less dynamic viral nucleosomes would hinder transcription). The hDaxx/ATRX complex, which mediates the assembly of H3.3 into transcriptionally repressed chromatin, is recruited to HSV-1 genomes early during infection [100,110,111]. HIRA depletion may indirectly promote the hDaxx/ATRX-mediated assembly of repressive H3.3 nucleosomes with HSV-1 DNA, before ICP0 eventually disrupts this assembly complex [100].

H3.1 is only assembled into viral nucleosomes concomitantly with HSV-1 DNA replication [64]. Assembly is accompanied by a decrease in the pool of free H3.1 [99], and an increase in the level of total H3 occupancy on HSV-1 DNA [64,70]. Assembly of viral chromatin during HSV-1 DNA replication is also critical. Depletion of a component of the Caf1 complex that assembles H3.1 containing nucleosomes during cellular DNA replication, Asf1b, inhibits HSV-1 DNA replication [112]. Asf1 regulates cellular DNA replication fork progression by mediating the nucleosome disassembly and re-assembly ahead of and behind it, respectively [113]. Similar activities may be required for efficient viral DNA replication. 

ICP0 targets the H3 variant CENP-A (and other centromeric proteins) for proteasomal degradation [80,114,115,116]. This degradation limits the potential for assembly of CENP-A nucleosomes with HSV-1 genomes. CENP-A is typically deposited in centromeric nucleosomes through replication-independent chromatin assembly [117]. The CENP-A chaperone, Holliday-junction recognition protein (HJURP), is recruited to centromeres to mediate CENP-A nucleosome assembly [118,119,120]. Artificial targeting of HJURP to non-centromeric loci is sufficient for assembly of CENP-A nucleosomes, which in turn is sufficient to recruit constitutive centromere-associated proteins, such as CENPs-N, -M, -T, and ‑C [119]. HJURP recognizes Holliday-junctions, which may well form during circularization or replication of HSV-1 genomes. Nucleosomes containing CENP-A have slower turnover rates than those containing H3 [117], decreasing nucleosome dynamics and facilitating silencing.

***Histone PTM, Acetylation.*** Histones are reversibly acetylated, by KAT (AKA histone acetyltransferases, HAT) and deacetylases (histone deacetylases, HDAC). Their acetylation neutralizes charge-dependent interactions with DNA and adjacent nucleosomes, promoting nucleosome turnover and increasing DNA accessibility.

The histones associated with HSV-1 genomes in lytically infected cells are enriched in H3K9ac and H3K14ac [50,121], PTM associated with active transcription and nucleosome instability. Consistent with such acetylation, the viral transcription transactivators VP16 and ICP0 recruit KATs and disrupt HDAC activity. VP16 recruits the KATs, GCN5, PCAF, and CBP/p300 to promote localized histone acetylation [60,86,106,122]. ICP0 also promotes histone acetylation through the stimulation of and interaction with PCAF, CBP/p300, and CLOCK, while disrupting HDACs [77,79,123,124,125]. Surprisingly, single or combinatorial knockdown of some of these KATs (CBP/p300, GCN5, PCAF) does not drastically affect IE gene transcription [69,89,126]. If anything, their depletion tends to enhance it [69,89,126]. However, global depletion of KATs would be expected to decrease the turnover of cellular nucleosomes. KAT-depleted cells likely have impaired nucleosome turnover before they are infected, and their ability to mobilize histones in response to infection is likely compromised, too. Viral gene expression would then have fewer obstacles to overcome in the knocked-down cells. Such effects on cellular nucleosome dynamics could very well account for the apparently paradoxical increase in IE gene expression in KAT depleted cells.

Global histone acetylation increases late during infection [50,121], but histones are mobilized to a basal degree before these changes occur [94,98]. The functional analysis of histone mobilization may be more sensitive than the morphological analysis of their PTM. Moreover, global evaluations cannot detect potentially relevant PTM in only the mobilized histones. The reduction in viral protein expression when CLOCK is depleted [125], for example, suggests that this KAT may have a more localized role during HSV-1 infection than p300, PCAF, or GCN5. The CLOCK/BMAL1 complex normally regulates circadian rhythms; it is consequently tightly regulated. CLOCK/BMAL1 is activated by SUMO-modification of BMAL1, which results in its localization to ND10s [127], ubiquitination, and proteasomal degradation [127]. However, ICP0 interacts with and stabilizes BMAL1 [125], stabilization which may promote localized CLOCK-mediated acetylation [128] of histones in the nuclear domains containing HSV-1 genomes. CLOCK may thus specifically promote viral nucleosome dynamics with little global effect on cellular nucleosome dynamics.

The interactions of ICP0 and VP16 with KATs and the dysregulation of HDACs indicate the important roles of these chromatin-modifying enzymes in the regulation of HSV-1 nucleosome dynamics and gene expression.

***Histone PTM, Methylation.*** Reversible histone methylation is catalyzed by lysine histone-methyltransferases (KMTs) and lysine histone-demethylases (HDM). Histone methylation is a complex mechanism with mono- to tri-methylation of specific lysine residues contributing to gene activation or repression. For example, histone H3 methylation on lysines 4 (H3K4me) or 36 (H3K36me) correlate with gene activation, whereas methylation on lysines 9 (H3K9me) or 27 (H3K27me) correlate with gene repression. Some methylations are mutually exclusive, such as H3K4me and H3K9me (fully so) or H3K4me and H3K27me (partially so). The effects of methylation on histone dynamics are less direct than those of acetylation, phosphorylation, or poly-ADP-ribosylation, as methylation does not alter histone-DNA charge interactions.

The histones associated with HSV-1 genomes early after infections were modified with the repressive H3K9me3. HCF-1 then recruits a regulatory complex containing Set1/MLL1 KMT and LSD1/ JMJD HDM to the viral IE promoters [61,102,103,104,129,130]. JMJD and LSD1 HDMs act in sequence to remove H3K9me3, such that the SET1/MLL1 KMT can then methylate H3K4 [61,68,102,103,104]. Removal of H3K9Me, and the subsequent methylation of H3K4, is critical for HSV-1 transcription and DNA replication [61,68,102,103,104]. Inhibition of JMJD or LDS1 increases histone occupancy and inhibitory PTM on the HSV-1 genomes, inhibiting HSV-1 transcription and DNA replication [68,103,104]. The accumulation of histones bearing repressive methylation in the absence of LSD1 or JMJD indicates that the replacement of H3K9me3 with H3K4me3 is critical in the regulation of HSV-1 nucleosome dynamics. Accumulation of H3K9me3 on HSV-1 chromatin also induces the subsequent association of HP1 and the formation of heterochromatin, further restricting viral nucleosome dynamics and promoting HSV-1 genome silencing.

SET7/9 methylation appears to hinder HSV-1 gene expression. SET7/9 generally methylates H3K4 to promote gene expression [131,132]. However, depletion of SET7/9 stimulates HSV-1 gene expression and replication [61]. This effect parallels the stimulation of HSV-1 gene expression in HIRA or KAT depleted cells. SET7/9 may therefore also function in the global regulation of nucleosome dynamics, as opposed to having only localized effects on HSV-1 nucleosomes. SET7/9 depletion may hinder the mobilization of histones from the cellular chromatin, which may in turn limit the availability of histones to silence viral gene expression.

***Histone PTM, Phosphorylation.*** Phosphorylation directly contributes to histone exchange and nucleosome dynamics by introducing negative charges, which weaken interactions with DNA. However, the phosphorylation of histones in HSV-1 or cellular chromatin during infection remains largely unknown. The sole histone phosphorylation directly examined is that of H2A.X (referred to as γ-H2A.X). H2A.X phosphorylation typically occurs in response to cellular DNA damage. H2A.X is also phosphorylated during HSV-1 infection [133], perhaps due to the activation of some components of the cellular DNA damage response. γ-H2A.X localizes to the cellular chromatin at the periphery of viral replication compartments [133].

Histone phosphorylation also regulates nucleosome turnover indirectly. Phosphorylation of H3T118 promotes nucleosome disassembly by SWI/SNF chromatin remodelling complexes [134]. H3T118P or H3T32P also have high affinity for the histone chaperone NAP [135], an interaction that precludes H3 binding to DNA [135]. Increased NAP association therefore likely stimulates nucleosome disassembly and turnover. Phosphorylation of H3T11 stimulates JMJD2C activity towards H3K9me2/me3, whereas phosphorylation of H3T6 prevents LSD1 demethylation of H3K4 and stimulates preferential demethylation of H3K9 [136]. By such mechanisms, H3 phosphorylation could also modulate the demethylations critical for initiation of viral IE gene transcription [68,103,104]. 

Histone phosphorylation also stimulates transcription through the reduction of silencing stimuli. For example, phosphorylation of H3S28 displaces the PcG (polycomb group) KMT that mediates gene silencing though the establishment of H3K27me3 [137], and H3S10P impairs HP1 binding to H3K9me3. Via such mechanisms, histone phosphorylation inhibits nucleosome stability and heterochromatin formation [138,139], disrupting gene silencing and promoting nucleosome turnover. Phosphorylation also promotes gene expression more directly. For example, H3S10P promotes the activating GCN5 acetylation of H3K14 at the promoters of transcribed genes [140].

Linker histones have several phosphorylation sites in their N- and C-terminal tails [141]. H1 directly stabilizes chromatin and promotes the formation of higher-order chromatin structures [141]. It also hinders core histone acetylation and nucleosome remodelling by ATP-dependent chromatin remodelers [142,143]. These effects reduce nucleosome turnover. H1 phosphorylation promotes its disassociation from chromatin [144], and H1 chromatin exchange is enhanced in infected cells [94]. H1 mobilization may be required to permit the mobilization of core histones, or core histone mobilization may promote that of H1. However, whether HSV-1 chromatin contains H1 has not been evaluated; nor has the contribution of H1 phosphorylation to its mobilization in infected cells.

***Histone PTM, Ubiquitination.*** All core histones are ubiquitinated, but ubiquitination of H2A and H2B is the most prevalent. Mono-ubiquitination of H2A is generally associated with transcription repression, whereas that of H2B contextually regulates transcription and chromatin structure. H2A and H2B are also ubiquitinated in response to DNA damage. HSV-1 encodes its own ubiquitin ligase, ICP0 [10,145,146], and thus modulates ubiquitination pathways. Although ICP0 mediated ubiquitination is well known to limit cellular antiviral responses, its effects on chromatin dynamics are not fully elucidated.

Monoubiquitination of H2A, on K119, is largely mediated by the E3 ligase component of polycomb repressive complex 1 (PRC1), RNF2 (Ring1 and Ring2). H2AK119ub facilitates the chromatin association of H1 [147], which in turn promotes the formation of higher-order chromatin structures to limit DNA access and repress gene expression [148]. H2AK119ub is sufficient to instigate chromatin compaction and gene repression, in that the PRC1- like 4 (PRC1L4) complex is recruited to chromatin independently of additional repressive PTM (such as H3K9me or H4K20me) [148]. H2AK119ub would limit HSV-1 nucleosome dynamics and promote silencing. Ubiquitination of H2A in HSV-1 nucleosomes has yet to be evaluated, as does the regulation and recruitment of the relevant E3 ligases.

The total nuclear levels of H2Aub decrease during lytic infection [149]. Such a global decrease would globally decrease nucleosome stability, promoting nucleosome turnover and increasing the availability of histones to silence viral genomes. Like H2B and H4, H2A is also mobilized during infection [150]. Viral mechanisms may also locally decrease H2A ubiquitination to specifically promote the turnover of HSV-1 nucleosomes.

Two E3 ubiquitin ligases, RNF8 and RNF168, typically catalyze the ubiquitination of H2A and H2A.X in response to cellular DNA damage. RNF8 localizes first to DNA lesions, but does not ubiquitinate nucleosomal H2A efficiently [151]. The formation of H2A polyubiquitin chains by RNF8 requires previous mono-ubiquitination on H2AK13-15 by RNF168 [151]. H2AK13-15ub is proposed to promote nucleosome exchange to facilitate DNA repair [151]. The H2A polyubiquitin chains recruit other proteins to the sites of DNA damage, including the E3 ligase BRCA1/BARD1 complex that further ubiquitinates H2A and H2B [152]. The decrease in ubiquitinated H2A (and H2A.X) during HSV-1 infection results from ICP0 mediated proteasomal degradation of RNF8 and RNF168 [81]. ICP0 also disrupts the redistribution of BRCA1 to the nuclear domains associated with HSV-1 genomes [153]. These activities of ICP0 suggest that H2A ubiquitination limits HSV-1 nucleosome dynamics to restrict viral gene expression. Under this model, the H2A in HSV-1 chromatin during latency or quiescence would be expected to be ubiquitinated. In contrast to its effects on RNF8 and RNF168, ICP0 does not alter the levels of RNF2 or 2A-HUB [81]. These ubiquitin ligases may thus still ubiquitinate a sub-population of H2A in the context of a global decrease in H2Aub.

Ubiquitination of H2BK120 (H2BK123 in yeast) stabilizes the nucleosome and promotes its assembly during transcription or DNA replication [154,155,156]. Nucleosomes with ubiquitinated H2B have slower turnover rates, resulting in apparently increased nucleosome occupancy [154]. Decreased H2Bub conversely enhances nucleosome dynamics, resulting in an apparent genome wide decrease in nucleosome occupancy [154,155] Ubiquitination of H2B in promoter nucleosomes inhibits transcription [155]. The slower turnover rates of H2Bub nucleosomes are proposed to prevent RNAPII binding or transcription complex assembly [154,155]. In contrast, ubiquitination of H2B in coding regions facilitates nucleosome reformation following passage of RNAPII, supporting transcription elongation [154,155]. H2Bub also stimulates the activating H3K4 and H3K79 methylations [154,157]. In contrast, H2Bub inhibits H3K36 methylation [155], a PTM enriched in transcribed genes (of yeast) which inhibits histone exchange during transcription [158]. The levels of ubiquitinated H2B decrease during infection independently of ICP0 [81]. ICP0 ubiquitination of a sub-population of H2B may promote HSV-1 transcription by regulating RNAPII transcription elongation.

Ubiquitination of H2B also promotes nucleosome reassembly during DNA replication and contributes to the progression of the replication fork [156]. In yeast, non-ubiquitinable H2B mutants destabilize the replisome, impairing replication fork progression [156]. They also have decreased H3 occupancy at the origins of replication and delayed H3 deposition during DNA replication [156]. The phenotype is consistent with defects in nucleosome formation, or stabilization, during chromatin assembly. Under such replication stress, homologous recombination (HR) dependent mechanisms may be activated to complete DNA replication [156]. The global decrease in ubiquitinated H2B during infection may contribute to the removal of nucleosomes from the HSV-1 genomes for encapsidation. Loss of ubiquitinated H2B in HSV-1 chromatin during DNA replication may impair DNA replication and activate HR pathways. Efficient HR repair of cellular double-stranded breaks (DSB) requires NBS1, ubiquitination of H2B by RNF20, and SNF2H [159]. These proteins induce chromatin remodelling and promote the eviction of nucleosomes adjacent to the DSB to facilitate DNA resection [159]. SNF2H and NBS1 localize to the HSV-1 replication compartments, where they may well enhance nucleosome dynamics during HSV-1 DNA replication or genome encapsidation by similar mechanisms. Unfortunately, the localization of RNF20 in infected cells is still unknown.

***Histone PTM, SUMOylation.*** SUMO modification contextually promotes transcriptional activation or repression. SUMOylation mediates the recruitment of chromatin-modifying factors. In turn, such factors modulate nucleosome dynamics to regulate transcription. Some of the SUMO-regulated repressive chromatin modifying-complexes, such as hDaxx/ATRX, CoREST/LSD1, and HDACs, as well as HP1 are important regulators of HSV-1 nucleosome dynamics. ICP0, a SUMO-targeted ubiquitin ligase (STUbL) [160], may very well disrupt the recruitment of repressive complexes to HSV-1 genomes by targeting essential SUMOylated proteins.

All core histones are SUMOylated [161,162,163]. Histone SUMOylation is generally associated with transcriptional repression [161,162], and opposes activating PTM, such as acetylation [162]. SUMOylation would thus be expected to impair nucleosome dynamics. However, SUMO-labeled H2B was associated with transcriptionally active euchromatin and excluded from heterochromatin [154]. Furthermore, SUMOylated H2B globally altered chromatin structure, enhancing nucleosome dynamics [154]. There is an increase in the levels of SUMO conjugated proteins during infection with ICP0 null HSV-1, and SUMO and SUMO-conjugating enzymes localize to nuclear domains adjacent to infecting HSV-1 genomes [160]. Histone SUMOylation may regulate HSV-1 nucleosome dynamics to promote or repress HSV-1 transcription. The SUMO-mediated recruitment of repressive complexes to the viral genomes, however, implicates this PTM in viral repression.

***Histone PTM, Poly-ADP-ribosylation.*** Proteins are reversibly modified with mono- to poly-ADP-ribose (PAR) moieties. Linker and all core histones have ADP-ribosylation sites [164]. Histone H1 is a major PAR substrate, with glutamate ADP-ribosylation sites in the amino (N)- and carboxyl (C)‑terminal tails and a lysine ADP-ribosylation site in the C-terminal tail. Core histones have one (H2A, H2B, H4) or two (H3) lysine ADP-ribosylation sites in their N-terminal tails. H2B has an additional glutamate modification site in its N-terminal tail. Typically, only a small percentage of histones are ADP-ribosylated at any given time. ADP-ribosylation adds a negative charge, which is compounded in poly-ADP-ribose chains. Histone ADP-ribosylation relaxes chromatin structure, potentially via charge-repulsions similar to those of acetylation. Consistently, ADP-ribosylation is associated primarily with transcriptionally active loci and chromatin that is extensively poly-ADP-ribosylated is more dynamic. For example, PARylation by the ADP-ribosyltransferase (ART) ARTD1 (PARP1) induces rapid transcription-independent loss of nucleosomes (H3) across the *Hsp70* gene after heat shock [165].

HSV-1 activates ARTD1, possibly as a result of activation of the DNA damage responses, globally increasing poly-ADP-ribosylation levels [166]. ARTD1, and the related ARTD5 (Tankyrase1), localize to the replication compartments [166,167,168]. However, their substrates, or the PARylation of histones, during infection are unknown. ADP-ribosylation of histones in HSV-1 nucleosomes would enhance their dynamics. Consistently with a role for PARylation in activating HSV-1 transcription, ICP0 triggers proteasomal degradation of the poly-(ADP-ribose) glycohydrolase (PARG) to promote the maintenance of PAR chains [166]. However, inhibitors of ADP-transferase activity only marginally decrease viral replication [166,168]. PARylation would likely also contribute to cellular nucleosome dynamics. As discussed, any inhibition of any mechanisms that mobilize histones to make them available to interact with viral genomes can lead to a variety of outcomes, depending on whether the modification plays a more prominent role in the viral or cellular nucleosomes.

***ATP-dependent chromatin remodelling.*** Chromatin remodelling complexes are large multi-subunit complexes that catalyze the ATP-dependent restructuring and repositioning of nucleosomes. The ATP‑dependent chromatin remodellers are classified into the SWI/SNF, ISWI, CHD, and INO80 families. The ISWI family mainly space nucleosomes via sliding. These complexes have roles in chromatin assembly, and transcription repression or activation. ISWI remodellers bind extrachromosomal DNA, with preference for longer DNA, to remodel and centre nucleosomes. Single-stranded DNA gaps, common in HSV-1 genomes, do not impede ISWI remodelling activity. SWI/SNF produce multiple outcomes including octamer ejection, dimer displacement and exchange, nucleosome sliding, and disome formation [169]. They also promote nucleosome exchange by facilitating octamer transfer in trans. Their BRG/BRM catalytic subunits have C-terminal bromodomains, which bind to acetylated histones and contribute to promoter targeting.

Chromatin remodellers from the ISWI (SNF2H) and SWI/SNF (BRG1, BRM, BAF155, BAF57, BAF170, and BAF60a) families interact with HSV-1 proteins and localize to the replication compartments [60,106,167]. SNF2H promotes HSV-1 gene expression and replication [105], and localizes to late replication compartments [167]. Knockdown of SNF2H decreases IE gene transcription, and viral replication, and increases H3 occupancy on HSV-1 promoters [105]. The apparent late reduction in H3 occupancy is SNF2H independent, however, suggesting that the largest effect of SNF2H is in the initial chromatin assembly and viral gene activation [105]. Consistent with this model, SNF2H is recruited by H3K4me-2 -3 [170], an important PTM in the activation of IE gene expression. VP16 recruits BRG and BRM to IE promoters [60,106]. BRG/BRM may also be recruited to acetylated HSV-1 promoters via bromodomains to regulate nucleosome dynamics during HSV-1 gene transcription. Depletion of BRG and BRM subunits unexpectedly tended to increase IE gene expression [69,89]. As other chromatin modifiers, however, BRG/BRM may well regulate the nucleosome dynamics in cellular chromatin as well. Their depletion would then impair the mobilization of histones from cellular chromatin, impairing the cellular antiviral responses that restrict viral replication. The available data suggest that chromatin remodelling by SNF2H is more relevant for HSV-1 nucleosome dynamics, whereas remodelling by BRG/BRM likely also regulates cellular nucleosome dynamics.

The mammalian family of INO80 chromatin remodelling ATPases exchange H2A for the variant H2A.Z. Nucleosomes containing H2A.Z, particularly in combination with H3.3, are highly unstable and promote chromatin relaxation and nucleosome exchange [33]. The NuA4 complex is recruited to cellular DSB, where the p400 ATPase component catalyzes the exchange of H2A with H2A.Z [171]. Subsequently, the Tip60 subunit mediates H4 acetylation [171]. The NuA4 complex thus initiates chromatin relaxation around the DSB to promote nucleosome dynamics and create a DNA template suitable for repair. The increased turnover of histones γH2A.X, H2A.X, and H3 within damaged chromatin requires the p400 ATPase activity and H2A.Z [171]. H2A.Z is mobilized during HSV-1 infection [150], although the presence of H2A.Z in HSV-1 chromatin or the activity of NuA4 during HSV-1 infection has yet to be evaluated. Tip60 is recruited to β- and γ-herpesvirus genomes, to stimulate lytic replication [172]. However, whether it is recruited as a component of the NuA4 complex or not was not evaluated. NuA4 is recruited to DSB independently of γH2A.X [171]. The NuA4 complex may thus also be recruited to HSV-1 DNA, which is devoid of γH2A.X, to mediate the exchange of H2A with H2A.Z in viral nucleosomes. The assembly of H2A.Z in HSV-1 nucleosomes would increase the instability of the viral chromatin and counteract cellular silencing attempts. However, H2A.Z and NuA4 would also be expected to regulate both cellular and viral chromatin dynamics. The potential effects of their depletions on HSV-1 gene expression, therefore, would depend on the balance of their activities on the cellular and viral chromatin.

## 4. Conclusions

The results of the original MCN protection and chromatin immunoprecipitation (ChIP) assays appeared to be incompatible. The former seemed to indicate that there were not enough nucleosomes on the HSV-1 genomes to regulate transcription (Figure 1). In contrast, the latter appeared to indicate that chromatin did regulate HSV-1 gene expression. It has become clear in the recent years that HSV‑1, and cellular, chromatins are highly dynamic. With this new understanding, the results from ChIP, MCN accessibility, FRAP, and biophysical fractionations are entirely consistent with a model in which infected cells attempt to silence the infecting HSV-1 genomes by establishing silenced chromatin, but viral proteins disrupt this silencing by mobilizing histones away from the viral genomes (Figure 2). However, the mechanisms whereby histones are mobilized away from the cellular genome, then assembled in chromatin with viral genomes, and then mobilized away from them remain to be elucidated. The analyses of these mechanisms are complicated because the same general mechanisms are likely involved in the regulation of cellular and viral chromatin dynamics. The available evidence suggests that the same cellular proteins, complexes, and enzymes are likely involved in mobilizing the histones away from the cellular genome, and in mobilizing them away from the HSV-1 genomes. The first mobilization would make the proteins available to silence the HSV-1 genomes, whereas the second would de-silence the HSV-1 genomes and activate HSV-1 transcription. Consequently, perturbations of the steady state chromatin dynamics have opposing effects. They increase (or decrease) the availability of silencing proteins at the same time that they increase (or decrease) the ability of HSV-1 to mobilize these proteins away from its genomes. Most likely, new techniques, ingenuity, innovation, and a little luck will all be needed to analyze the mechanisms of silencing and antisilencing that govern the regulation of viral gene expression by chromatin. 
